# Multifractal Spectrum Curvature of RR Tachograms of Healthy People and Patients with Congestive Heart Failure, a New Tool to Assess Health Conditions

**DOI:** 10.3390/e21060581

**Published:** 2019-06-11

**Authors:** Ana María Aguilar-Molina, Fernando Angulo-Brown, Alejandro Muñoz-Diosdado

**Affiliations:** 1 Departamento de Física, Escuela Superior de Física y Matemáticas, Instituto Politécnico Nacional, Edif. No.9 U.P. Zacatenco, Mexico City 07738, Mexico; 2 Unidad Profesional Interdisciplinaria de Biotecnología, Instituto Politécnico Nacional, Av. Acueducto s/n, Barrio la Laguna, Ticomán, Mexico City 07340, Mexico

**Keywords:** multifractal, Chhabra and Jensen method, curvature, electrocardiogram, RR tachogram, congestive heart failure, NYHA index

## Abstract

We calculate the multifractal spectra of heartbeat RR-interval time series (tachograms) of healthy subjects and patients with congestive heart failure (CHF). From these time series, we obtained new subseries of 6 h durations when healthy persons and patients were asleep and awake respectively. For each time series and subseries, we worked out the multifractal spectra with the Chhabra and Jensen method and found that their graphs have different shapes for CHF patients and healthy persons. We suggest to measure two parameters: the curvature around the maximum and the symmetry for all these multifractal spectra graphs, because these parameters were different for healthy and CHF subjects. Multifractal spectra of healthy subjects tend to be right skewed especially when the subjects are asleep and the curvature around the maximum is small compared with the curvature around the maximum of the CHF multifractal spectra; that is, the spectra of patients tend to be more pointed around the maximum. In CHF patients, we also have encountered differences in the curvature of the multifractal spectra depending on their respective New York Heart Association (NYHA) index.

## 1. Introduction

A complex system has many components that interact with each other; it exhibits collective behavior and, due to the exchange of energy or information with the environment, can modify its internal structure and patterns of activity [1], but the interaction with environment is not the only mechanism that leads to complexity, for instance, it was shown both trough data analysis and modelling that the scaling and nonlinear multifractal features are intrinsically generated by the feedback mechanisms of cardiac control that act over a range of different time scales [2,3,4]. When we continuously measure the variables that describe a complex system, we usually obtain time series also with complex behaviour. Very often, these time series have monofractal or multifractal behaviour. Monofractal signals are more homogeneous that multifractal signals and they can be characterized by a single global scaling exponent, while multifractal signals require many exponents to fully characterize their scaling properties and they are intrinsically more complex and inhomogeneous [5,6,7,8,9,10].

After that the first findings of multifractality in physiological dynamics were reported by Ivanov et al. [11,12], multifractal analysis has been extensively used to study time series obtained from physiological systems [6,13,14,15], and many other kinds of complex systems with emergent properties such as urban systems [10], fuel mixtures in internal combustion engines [16], critical fluctuations in magnetic-field driven random systems [17], the self-organized social dynamics [18] and the fluctuations of stock market data [1], to mention a few. For instance, beat-to-beat RR interval time series are inhomogeneous and non-stationary; they fluctuate in an irregular and complex manner, suggesting that different parts of the signal have different scaling properties [8,11,12,13,19,20], including scaling differences associated to sleep-wake, sleep stages and even to circadian phases [21,22,23,24,25]. The multifractality of the heartbeat time series allows us to quantify the greater complexity of healthy dynamics compared to pathological conditions [8,11,15,20,26,27]. Multifractal analysis reveals a new level of complexity by the broad range of exponents necessary to characterize the healthy dynamics [28]. Additionally, fractal analysis (mono-and-multi-) can be used to test stochastic models that grossly reproduce the dynamics of beat-to-beat cardiac cycles [1,2,20,29,30].

Currently, heart diseases are becoming more common. These are a risk because if heart disease does not receive timely treatment, people would have serious complications. One of these complications is called CHF. The CHF occurs when the heart is weakened by diseases or conditions that damage the heart muscle [31,32,33]. The NYHA (New York Heart Association) classification is commonly used by cardiologists as a method to classify the severity of CHF. This classification has four values. NYHA I-patients do not have limitation of physical activity. NYHA II-patients have slight limitation of physical activity. They feel fatigue, palpitations, dyspnea (shortness of breath) when they do physical activity. NYHA III-patients have marked limitation of physical activity, i.e., less than ordinary activity causes fatigue, palpitations, or dyspnea. NYHA IV-patients have symptoms of heart failure at rest and when they do physical activity, the discomfort increases [31,32]. Heart rate data of subjects with a pathological condition, as CHF, show a clear loss of multifractality, indicating a near monofractal behaviour [8,11,15,20].

In this article, we apply the multifractal methodology to the analysis of RR tachograms of healthy people and patients with CHF (classification NYHA I to NYHA IV). We calculate multifractal spectra and observe in the resulting graphs that they tend to have a peak at the maximum in the case of CHF patients and that usually multifractal spectra of healthy subjects are right skewed while the majority of CHF spectra are left skewed or almost symmetric. For these reasons, we suggest to measuring the curvature around the maximum of the spectrum graphs and to characterize the symmetry of multifractal spectrum. These new parameters help us to differentiate time series of healthy subjects and CHF patients, but the most important fact is that these parameters give us information of the health status of CHF patients. The article is organized as follows: In Section 2 we introduce the used methods; in Section 3 we describe the databases; in Section 4 we present and discuss the results of our study and finally in Section 5, we present our conclusions.

## 2. Methods

### 2.1. The Multifractal Spectrum Width and Symmetry

To calculate the multifractal spectra, we use the method proposed by Chhabra and Jensen [34]. For completeness reasons, here, we present a summary of this method. We can consider time series as a singular measure *P(x)* if we normalize it. We obtain the fractal dimension *f(α)* covering the measure with boxes of length $L=2^{-n}$ and computing the probabilities $P_{i}\left(L\right)$ in each of the boxes. We then construct a one-parameter family of normalized measures $\mu_{i}\left(q,L\right)$, where the probabilities in the boxes of size *L* are [34]

(1) $$\mu_{i}\left(q,L\right)=\frac{{\left[P_{i}\left(L\right)\right]}^{q}}{\sum_{i}{\left[P_{i}\left(L\right)\right]}^{q}}$$

The fractal dimension is

(2) $$f\left(q\right)=\underset{L\to 0}{\lim}\frac{\sum \mu_{i}\left(q,L\right)log\left[\mu_{i}\left(q,L\right)\right]}{logL},$$

and the singularity strength is

(3) $$\alpha \left(q\right)=\underset{L\to 0}{\lim}\frac{\sum \mu_{i}\left(q,L\right)log\left[P_{i}\left(L\right)\right]}{logL}$$

Equations (2) and (3) provide a relationship between the dimension f(α) and the singularity strength α as implicit functions of the parameter *q*. To obtain multifractal spectra, for each *q* value, we evaluate the numerators on the right hand sides of the Equations (2) and (3), for decreasing box sizes (increasing *n*) and we plot these results versus *log L*. The obtained graphs are straight lines and we calculate the slopes and so we obtain a pair of coordinates *f(q)* and *α(q)* for each *q*. In this way we built the multifractal spectra.

The parameter *q* provides a microscope for exploring different regions of the singular measure. For *q* > 1, μ(q) amplifies the more singular regions of *P*, while for *q* < 1 it accentuates the less singular regions, and for *q* = 1 the measure μ(1) replicates the original measure [34], but from the point of view of the physiological interpretation and following to Amaral et al. [4] the *q* > 1 part of spectrum reflects singularities associated with large amplitude fluctuations in heart beat intervals, while the *q* < 1 part of the multifractal spectrum reflects singularities related to small amplitude fluctuations. This is directly related to neuro-autonomic regulation: during sleep the parasympathetic tone (PS) is dominant, which leads to large amplitude fluctuation in heart beat intervals; during wake the sympathetic tone (SS) is dominant, and heart beat dynamics exhibit much smaller fluctuations [4].

We used the Chhabra and Jensen method to obtain the multifractal spectra because this method calculates directly *f(α)* and it does not use the Legendre transforms [6,34]. The multifractal spectrum is a downward concave curve that gives us information about time series of complex systems.

We characterize the multifractal spectrum by its width and asymmetry. $\Delta \alpha =\alpha_{max}-\alpha_{min}$ is a measure of how wide the range of fractal exponents found in the signal; and, thus, it measures the time series degree of multifractality [8,11,12,19,20,26,27]. $\alpha_{0}$ corresponds to the maximum of f(α). The asymmetry depends on $\alpha_{max}$, $\alpha_{min}$ and $\alpha_{0}$. We define $\Delta \alpha_{right}=\alpha_{max}-\alpha_{0}$ and $\Delta \alpha_{left}=\alpha_{0}-\alpha_{min}$. If $\Delta \alpha_{right}=\Delta \alpha_{left}$, the spectrum is symmetric, and if $\Delta \alpha_{right}\neq \Delta \alpha_{left}$, it is asymmetric. If $\Delta \alpha_{right}>\Delta \alpha_{left}$, the spectrum is biased to the right and if $\Delta \alpha_{right}<\Delta \alpha_{left}$, the spectrum is biased to the left.

We introduce the symmetry parameter *r*, to better quantify the symmetry,

(4) $$r=\frac{\alpha_{max}-\alpha_{0}}{\alpha_{0}-\alpha_{min}}=\frac{\Delta \alpha_{right}}{\Delta \alpha_{left}}$$

**Figure 1:**
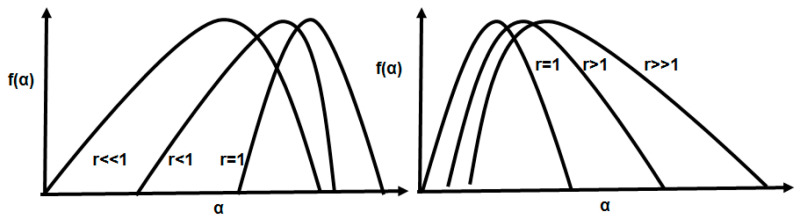
Different possible values of the symmetry parameter.

If *r* = 1 then the spectrum is symmetric, if *r >* 1 the spectrum is right skewed and if *r* < 1 then it is left skewed. If *r* << 1, the multifractal spectrum is sharply skewed towards the left. If *r* >> 1, the multifractal spectrum is sharply skewed towards the right (Figure 1).

The change in shape of the curve for the CHF group may provide insights into the alteration of the cardiac control mechanisms due to this pathology and gives information about the morphology of the multifractal spectrum around the maximum.

### 2.2. Curvature

At any point of the graph of a function y=f(x), the curvature can be calculated using the relationship [35,36]:

(5) $$K=\frac{\left|\frac{d^{2}y}{dx^{2}}\right|}{{\left[1+{\left(\frac{dy}{dx}\right)}^{2}\right]}^{3/2}}$$

**Figure 2:**
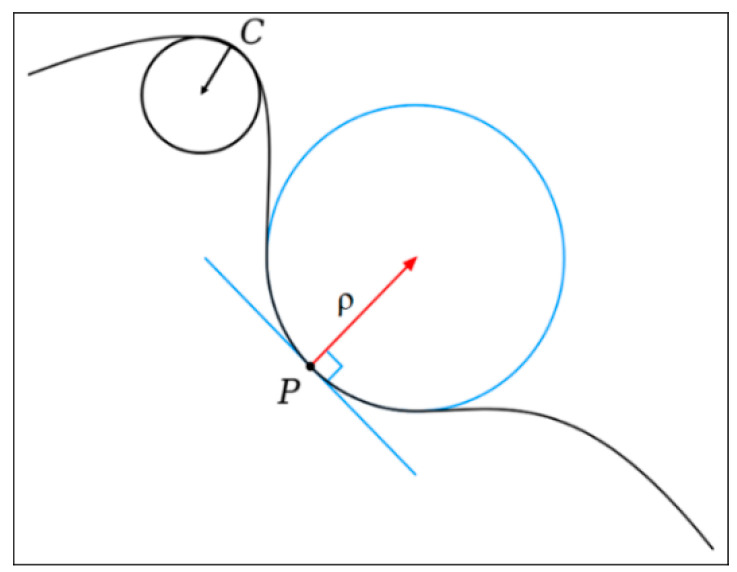
The curvature is defined in terms of the inverse of the radius of the circle of curvature. The curvature around point P is smaller than the curvature around point C.

As illustrated in Figure 2, at point C the curvature of the graph of the function is greater than at point P, the circles shown are called circles of curvature and *ρ* is the radius of such circles and is called the radius of curvature, the curvature is calculated as K=1/ρ. At a point where the function is almost flat, the curvature is practically zero and at points where the function has almost a peak the curvature will be very large.

In the case of multifractal spectra, around the maxima we can expect great values of curvature and the curvature must be close to zero for large values of |q|. Recently, the curvature of the multifractal spectrum was used to estimate to the degree of fractality of the temporal evolution of London’s street network from 1780 to 2010 [10].

## 3. Databases

Heartbeat time series were analysed for healthy people and CHF patients. The databases were obtained from the Physionet website [37]; these are 24 h recordings of heartbeat time series, taken by ambulatory recorders (Holter). 

We downloaded three different databases. The first database is called Normal Sinus Rhythm (nsr) RR Interval Database it has data from 54 healthy subjects (30 men, age 28–76, and 24 women, age 58–73). The second and third databases have CHF patients. The second database is called Congestive Heart Failure RR Interval Database and contains information of 29 CHF patients (34–79 years) including information of their NYHA classification (class I-III). The third database is called the BIDMC CHF Database and it contains 15 patients (11 men of 22–71 years and four women 54–63 years) with severe CHF (NYHA class III-IV). That is, we have 44 CHF patients. We analysed these 24 h-time series and then we obtained two segments of six hours from each time series: one when the subjects were asleep and the other when the subjects were awake. However, we only analysed 40 of these 6 h CHF segments when the patients are asleep and 38 when they are awake. The cases of 6 h CHF not considered, are not reported because it was not possible to have continuous periods of 6 h either asleep or awake.

## 4. Results

**Figure 3:**
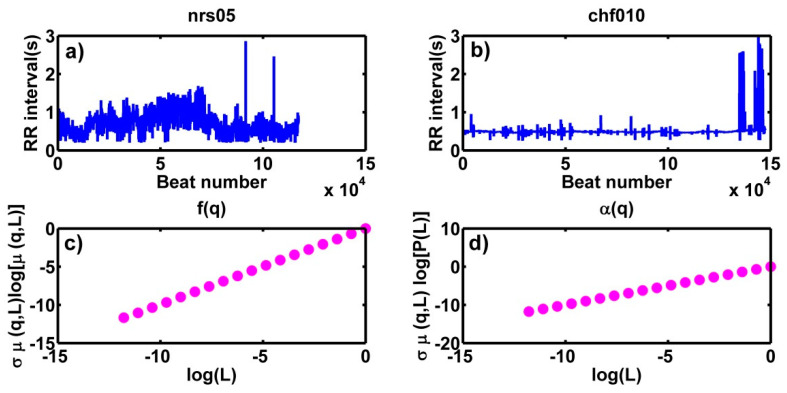
Two segments of 24 h-tachograms of a healthy subject (**a**) and a CHF patient (**b**). Graphs (**c**) and (**d**) show the graphs that are constructed to obtain α(q) and f(α (q)) for the case q = 9.

For each heartbeat time series, we calculated their multifractal spectra and measured $\alpha_{max}$, $\alpha_{min}$ and $\alpha_{0}$; the width of spectra ∆α, and the symmetry parameters $\Delta \alpha_{rigth}$, $\Delta \alpha_{left}$ and *r*. The calculations were made from q=−10 to q=10, with bins of 0.1 [11,12]. Figure 3 shows two typical segments for a healthy person (nsr05) (a) and a patient with CHF (chf010) (b). For each *q*-value and following the described method of Chhabra and Jensen [34], the plots indicated in (c) and (d) are made to determine α(q) and f(α(q)) by using least squares; it is shown in the case where q = 9, that the multifractal spectrum is constructed with the pairs (α(q), f(α (q)).

It has been reported that Δα values of multifractal spectra decrease with disease and age [11,12,15,19,20] and there are important reported results relative to the comparison between asleep and awake subjects regarding the width of the multifractal spectra [21,22,38]. In this work, we focus our study on the symmetry and shape of the spectra in order to obtain new parameters to better differentiate between healthy subjects and CHF patients. Similarly, we want to find differences between each NYHA classification; it means that we want to discriminate the severity of the disease. For instance, we hope that multifractal spectra of NYHA I-CHF patients would be very different of the multifractal spectra of NYHA III-IV-patients. In order to improve this task, we analyse the total series (24 h) and subseries (6 h) when the patients were asleep and other subseries (6 h) when the patients were awake.

### 4.1. On the Symmetry of the Spectra

As observed in Table 1, the spectra of healthy subjects tend to be right skewed; in fact, when we analysed the 24 h total series, 49 (90.7%) spectra were right skewed and five were left skewed. However, the analysis of the 6 h time series when the subjects were asleep showed that the 54 (100%) spectra were right skewed. In addition, analysis of the 6 h time series when these healthy subjects were awake showed that 45 (83.3%) spectra were right skewed and nine spectra were left skewed. In contrast, in the spectra of CHF 24 h time series we found that 25 spectra (56.8%) were right skewed and 19 spectra were left skewed. Nevertheless, the analysis of the 6 h time series when the CHF patients were awake showed that 21 (55.3%) spectra were right skewed and 17 spectra were left skewed. In contrast, analysis of the 6 h time series when the CHF patients were asleep showed that 31 (77.5%) spectra were right skewed and nine spectra were left skewed (Table 1). 

We calculated *r* as a better way to quantify the symmetry. In both cases (healthy and CHF) we have right (*r >* 1) and left skewed spectra (*r* < 1). The difference is that we observed that many of the healthy spectra that are right skewed are sharply biased to the right (*r* >> 1) and the spectra that are left skewed are slightly left skewed. In general, CHF patient spectra are slightly right skewed, the rest are left skewed and some of them are sharply left skewed (*r* << 1).

For instance, the maximum value of *r* for the 24 h time series of healthy subjects is the nsr30 case (nsr is for normal sinus rhythm), we found for this subject *r_max_* = 6.7 (see Table 2), this is a multifractal spectrum very right skewed. There are some multifractal spectra of healthy subjects that have *r* values slightly less than 1 and they are slightly left skewed, as for example, for one 24 h-time series of a healthy male person (nsr50), we obtained *r_min_* = 0.9 (Table 2). It means that the multifractal spectrum is slightly asymmetric and left skewed.

For 6 h time series of sleeping subjects, we found that all the 54 spectra are right skewed, for example, the maximum value of *r* is the nsr15, we obtained for this subject an *r* value of *r_max_* = 31.9 (Table 3), this is a spectrum sharply biased to the right. For 6 h time series of awake subjects we found that the maximum value of r is *r_max_* = 4.1 which corresponds to the subject nsr24. The minimum *r* value was *r_min_* = 0.7 (Table 3) for nsr05.

Considering the 24 h-time series spectra of CHF patients, the maximum *r* value belongs to the chf008 patient, that has *r_max_* = 2.5 (Table 4). It means that the multifractal spectrum is right skewed, but it is not as right skewed as the nsr30 multifractal spectrum. The minimum *r* value belongs to the chf012 patient, *r_min_ =* 0.4 (Table 4), this *r* value is associated with a spectrum sharply biased to the left.

For the 6 h time series of sleeping CHF patients we found that the maximum *r* value belongs to the chf203 patient, *r_max_* = 3.7 (Table 3), while the minimum corresponds to the chf012, it is *r_min_* = 0.2 (Table 3). For 6 h time series of awake CHF patients we found that the maximum value of *r* is *r_max_* = 3.6 (Table 3) which corresponds to the subject chf008. The minimum *r* value was *r_min_* = 0.3 (Table 3) for chf013. In both cases the minima values indicate that the spectra are sharply biased to the left.

Based on the results we can conclude that right skewed spectra are associated to healthy subjects, especially when they are sleeping, the 100% of the spectra were right skewed in this case. Although the CHF patients present right and left skewed spectra; in general, the right skewed spectra of CHF patients are not as right skewed as the spectra of healthy subjects for the three cases, 24 h, 6 h asleep and 6 h awake; besides the left skewed spectra of CHF patients are in general more left skewed that the spectra of healthy subjects for the three cases. Special attention should be paid in Table 1 to the case of sleeping subjects and patients, because there is a big difference between the *r* average values in this case.

**Figure 4:**
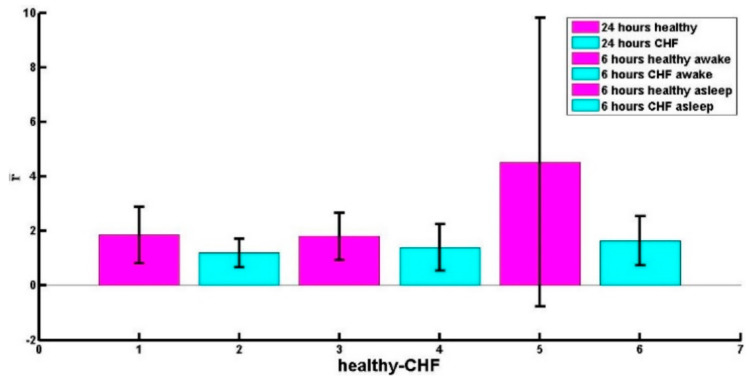
Bar chart showing the average values of *r*, mean *r*-values are compared for series of healthy adults and patients with CHF. The statistical differences were significant for the 24 h (*p* ≈ 0.001), 6 h awake (*p* ≈ 0.025) and 6 h asleep (*p* ≈ 0.00025) tachograms. The big error bar for the case of awake 6 h series is due to the fact that there are many cases (see Table 3) with spectra sharply skewed towards the right.

Statistical tests were made to verify if there was a significant difference between the average values of *r*, for example in Figure 4, a bar diagram is shown comparing the average values of *r* for the 24 h series and the 6 h series in the two cases analysed, awake and asleep, in all three cases the differences were statistically significant. In all the statistical tests that we made in this article we used a significance level of 0.05.

### 4.2. On the Curvature of the Spectra around the Maximum 

**Figure 5:**
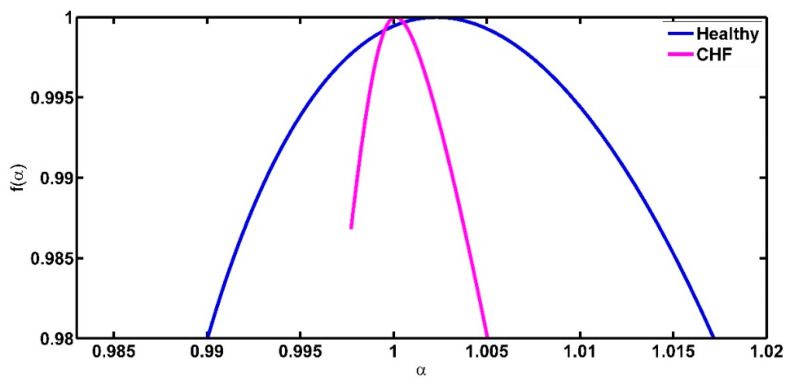
Multifractal spectra of one healthy subject (black) and one CHF patient (magenta). The curvature around the maximum spectrum is larger for the patient’s spectrum. That is, the spectrum of the patient has a spiky form in the maximum.

We note that the multifractal spectra of CHF patients have a spiky form around the maximum and the multifractal spectra of healthy persons tend to be most rounded around the maximum (Figure 5). We calculate the curvature of the multifractal spectra by using Equation (4); we numerically obtain first and second derivatives that we use to calculate the curvature. At the end we focus only on the curvature around the spectrum maximum because the curvature is almost zero in regions far from the maximum. The concept of curvature related to multifractality had been suggested by Ivanov et al. (2001) [8], when they affirmed that “the constantly changing curve of the *τ*(*q*) (the scaling exponents) curves for the healthy records suggest multifractality”. However, as far as we know, in our article it is the first time that the curvature concept around the maximum of the multifractal spectrum has been used to characterize the spectra of healthy people and patients with CHF, although the curvature of the multifractal spectrum has been used in other applications [10].

We constructed each multifractal spectrum as a set of points (α, f(α)), one point for each *q* value. As the calculation was made for *q* = −10 to *q* = 10, with bins of 0.1, at the end our multifractal spectra have 201 points. To calculate the first derivative we can use the forward difference *D_+_(h):f’(α)~D_+_(h) = (f(α + h) − f(α))/h* or the backward difference *D_−_(h):f’(α)~D_−_(h) = (f(α) − f(α − h))/h* and then we can combine both differences to estimate the second numerical derivative as [38,39,40]:

(6) $$f\prime \left(\alpha \right)\sim \frac{D_{+}\left(h\right)-D_{-}\left(h\right)}{h}=\frac{f\left(\alpha +h\right)-2f\left(\alpha \right)+f\left(\alpha -h\right)}{h^{2}}$$

**Figure 6:**
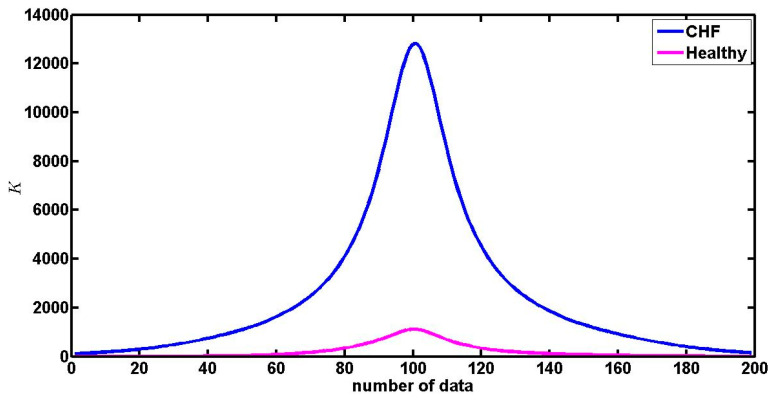
The curvature of the patient chf010 and the healthy subject nsr35, we are only interested in the maximum of this curve. As can be observed, the curvature around the maximum of the spectra of healthy people has lower values than the curvature of the spectra of the patients.

We used the first and second numerical derivatives to obtain the curvature by using Equation (5); we obtained plots of the curvature for the entire spectrum as is depicted in Figure 6, this figure is constructed with 199 points (the original plot has 201 points, the first derivative has 200 points and the second derivative has 199 points), the figure illustrates that the curvature around the maximum of the spectrum is big and it decreases almost to zero for big values of |q|. In this figure and in all figures that depict curvature, the notation “number of data” in the horizontal axis means that the first point corresponds to the curvature around the point of the spectrum that corresponds to *q* = −10, the last point corresponds to the curvature value around the point of the spectrum that corresponds to *q* = 10, then the central point (the number 100, that is the maximum) corresponds to the curvature around *q* = 0.

**Figure 7:**
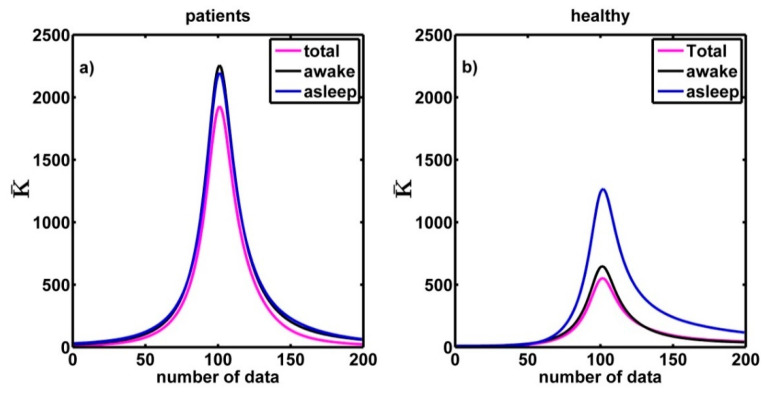
(**a**) Average curvature for three time series: 6 h-awake, 6 h-asleep and (total) 24 h. (**b**) The same for healthy subjects.

The curvature values were calculated for each one of the multifractal spectra (healthy people and patients with CHF for both 24 h- and 6 h-time series). We obtained the curvature plot (Figure 7) for each spectrum and then we obtained the average curvature plot for each three groups. Figure 7 shows the average curvature of the 24 h and 6 h (asleep and awake) times series for the CHF patients (Figure 7a) and healthy subjects (Figure 7b). The vertical axis represents the average curvature values. We notice in Figure 7a that the maximum value of the average curvature (mvac) for the CHF patients is 1724 ± 330 and in Figure 7b we see that for healthy persons mvac = 506 ± 92 (in both cases for the 24 h-time series). The difference is statistically significant, with a level of significance of 0.05 ($p\approx 2\times 10^{-9}$). On the other hand, for the case of 6h-time series (awake) the mvac for CHF patients is *1971 ± 377* and for healthy persons mvac = 587 ± 108, again the difference is statistically significant ($p\approx 4\times 10^{-11}$). For the case of asleep 6 h-time series mvac = 1986 ± 380 for CHF patients and mvac = 1177 ± 220 for healthy people, and again the difference is statistically significant (p≈0.0006). By comparing these values, we can see that the maximum average curvature of CHF patient is almost three times that of the healthy people when they are awake, but it is two times the mean curvature of healthy people when they are asleep. We can confirm that the multifractal spectra of patients tend to have the sharpest peak around the maximum.

In addition, Figure 7a reveals that the mvac value in the 6 h spectra when CHF patients were asleep or awake have similar values. We think that it occurs because the CHF patients have sleeping troubles and they are awake a lot of time or maybe it is because the condition of CHF patients is aggravated when they are sleeping [41]. Actually, if we make a comparison of the values of curvature around the maximum between healthy asleep and healthy awake we found that there is a significant difference between the corresponding means ($p\approx 8\times 10^{-8}$), but if the comparison is made between the values corresponding to sleeping congestive and awake congestive there is no significant difference (p≈0.91).

### 4.3. The Symmetry and the Curvature

When we calculate parameter *r*, we see that all the 54 healthy spectra are biased to the right when the subjects are asleep, and when we analyse 24 h-total time series spectra, five of them are slightly biased to the left and nine spectra corresponding to awake subjects are also slightly biased to the left. In the literature it has been reported that the spectra of healthy subjects are wide [8,10,13,15,16]. And in addition, from our results we observe that healthy subjects’ spectra tend to be biased to the right. On the other hand, we have found that the curvature of the spectra around the maximum is much larger for those corresponding to CHF-patients that for healthy subjects. It seems that this criterion can help to distinguish between CHF and healthy persons.

**Figure 8:**
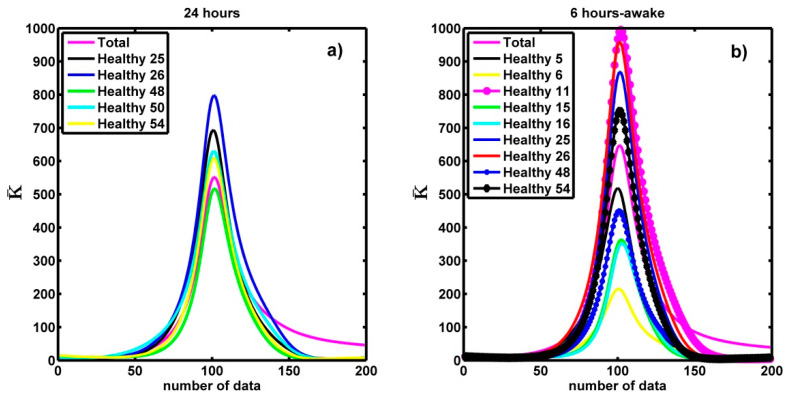
(**a**) Comparison of the average curvature for the 24 h time series of the healthy subjects with the average curvature values of the 5 spectra skewed to the left. (**b**) Comparison of the maxima of the average curvature for 24 h-time series of healthy persons with the maxima of the average curvature values of the 9 spectra skewed to the left in healthy people when they were awake.

In this section, by using the curvature, we examine the series of healthy people whose spectra were skewed to the left. We measure the curvature parameter values for these spectra. In Figure 8a, we show that the maximum values of the average curvature for 5 spectra of the 24 h time series are between 477 ± 86 and 724 ± 137 and they are similar to the average curvature value (506 ± 92) of healthy persons. We notice that those values are smaller than the maximum value of the average curvature for the spectra of 24 h-time series of CHF patients ($\bar{K}$ = 1724 ± 330).

We observe the same behaviour in the curvature of the spectra of the 6-h time series of the healthy subjects when they are awake (Figure 8b). Therefore, although these spectra were slightly left skewed, their curvature is like the average curvature of healthy subjects. Summarizing: although these spectra are slightly left skewed and we can think that they have cardiac problems, the curvature values are in the same range that the average for healthy subjects. Therefore, we have three criteria to say if a person is healthy, (a) their spectrum is wide, (b) their spectrum is loaded to the right and (c) the curvature around the maximum is small compared to the curvature of the CHF patient spectra. In contrast, for a CHF patient (a) the spectrum is narrow, (b) it is loaded to the left or slightly loaded to the right and (c) the curvature around the maximum is large.

### 4.4. The Curvature and the NYHA Classification

In this section, we study the average curvature of CHF patients according to the NYHA index. We use 24 h time series and 6 h-time series of two types: awake or asleep. In the previous section we obtained the average curvature for all the CHF patients. The average curvature was calculated for 24 h time series and we also calculated the average curvature for two groups (awake or asleep) of 6 h time series. We grouped the curvature values according to the NYHA index. These calculations were made to look for possible correlations between the curvature and the NYHA index.

Firstly, we classified the curvature data of the CHF patients of 24 h and 6 h (when they were asleep or awake) time series for each one of the NYHA classification and we found their maximum curvature values and we did a graph with these values of the average curvature. Then we compared the maximum values of the average curvature of each NYHA classification with the maximum values of the average curvature of the 24 h time series for healthy persons and for CHF patients.

**Figure 9:**
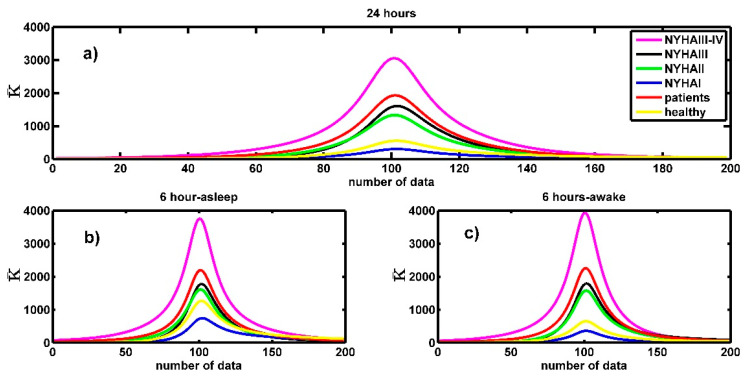
Comparison of the maximum values of the average curvature for: (**a**) 24 h-time series of the CHF patients with healthy persons with the maximum values of the average curvature according each NYHA classification. We observed that the curvature values increase with the damage in the heart, as average curvature maximum values in NYHA II, NYHA III and NYHA III-IV classification are close to the average curvature maximum values the CHF patients. In contrast, NYHA I classification has almost the value of healthy persons. (**b**) 6 h time series of the CHF patients and healthy persons with the average curvature maximum values of the 6 h time series of the CHF patients for NYHA classification when they are awake. We can see that the average curvature values increase with damage in the CHF heart disease. Also NYHA II, NYHA III, and NYHA III-IV average curvature maxima values are similar to CHF patient values and NYHA I has a similar value with healthy subjects. (**c**) 6 h time series of the CHF patients and healthy persons with the average curvature maximum values of the 6-h time series of the CHF patients followed NYHA classification when they are asleep. We can see that the average curvature values increasing with hurt in the CHF heart disease. Also, NYHA II, NYHA III, and NYHA III-IV average curvature maximum values are similar with CHF patients’ values, and NYHA I have similar values with healthy subjects.

In Figure 9a, we show the average curvature of the 24 h time series of the CHF patients for each NYHA classification. The vertical axis represents the average curvature values. It seems to be that the maximum curvature increases as the NYHA index increases, that is, it increases as the disease worsens. The average curvature maximum value in NYHA I is 278 ± 49 (see Figure 9a blue line). It is close to the maximum average curvature 506 ± 92 of the healthy subjects (see Figure 9a yellow line) in the 24 h time series. The maximum curvature values that follow are NYHA II 1175 ± 317 (see Figure 9a green line), NYHA III 1478 ± 270 (see Figure 9a black line), and NYHA III-IV 2680 ± 532 (see Figure 9a magenta line). These values are close to the average curvature maximum value 1724 ± 330 of the CHF patients in the 24 h time series (see Figure 9a red line). In 24 h tachograms, comparing the data of the curvature around the spectra maxima of healthy subjects with NYHA I CHF patients, no significant difference is obtained. The same happens when comparing the data of NYHA II CHF patients with NYHA III, but the difference between patients with NYHA I and NYHA II is significant ($p\approx 7.1\times 10^{-11}$), the difference between NYHA III and NYHA III-IV is also statistically significant ($p\approx 1.7\times 10^{-6}$).

In Figure 9b,c, we show the results that we have obtained in the 6 h time series when the CHF subjects are awake or asleep for each NYHA classification. As previously mentioned, the horizontal value represents the number of data that is obtained when we calculate the curvature numerically, i.e., it is a new horizontal axis with 199 points. We noted the same behaviour in these time series that for the 24 h time series. In Table 5 we show the average curvature maximum values that we obtained for all-time series, we noticed that the biggest curvature values correspond to NYHA II, NYHA III and NYHA III-IV. However, the average curvature maximum value in NYHA I classification has almost the same value that healthy persons, this is so because in the NYHA I CHF-patients the symptoms are present, but do not present discomfort and they can do almost anything as the healthy persons. In the 6 h awake tachograms, again there are statistically significant differences between NYHA I and NYHA II ($p\approx 7.4\times 10^{-5}$), and NYHA III and NYHA III-IV ($p\approx 2.6\times 10^{-7}$). And the same situation is repeated in the tachograms of 6 sleeping hours, there are statistically significant differences between NYHA I and NYHA II ($p\approx 1.6\times 10^{-5}$) and between NYHA III and NYHA III-IV ($p\approx 1.3\times 10^{-9}$). We did not find significant differences between the tachograms of patients with NYHA II and patients with NYHA III.

**Figure 10:**
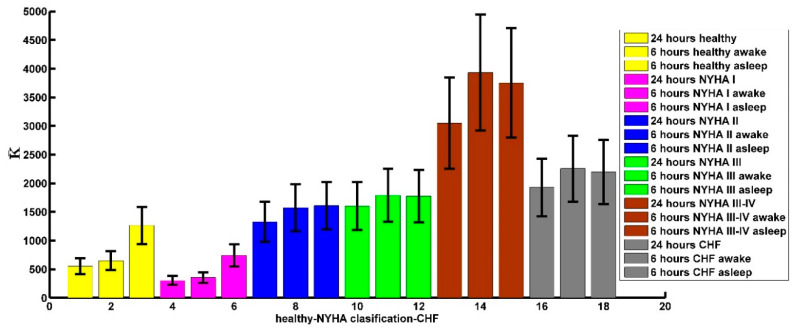
Bar chart for the values of the curvature around the maximum of the spectra of the tachograms of 24 h, 6 h awake and 6 h asleep. The significant differences between healthy and CHF are evident in all cases (24 h, 6 h awake and 6 h asleep). Also, between NYHA I and II and NYHA III and NYHA III-IV. And it is also observed that there are no significant differences between NYHA I and healthy and between NYHA II and NYHA III.

Figure 10 shows a bar diagram in which much of the information described in the previous paragraphs is summarized, as can be seen there is an important difference between the values of the average K between healthy and congestive. The differences already described between NYHA I and NYHA II and between NYHA III and NYHA III-IV can also be visualized in all cases. It is also observed that the heights of the bars of NYHA II and NYHA III cases are similar in all cases.

## 5. Conclusions

It has been reported that the multifractal spectra of healthy subjects tend to be wide and that the spectra tend to narrow with the diseases. We propose in this work that the spectra of healthy subjects tend to be right skewed and the curvature around the maximum of the spectra is small. Although different skewness of the multifractal spectra in healthy and CHF subjects was already shown in references [11,12], the focus of those articles was different. The multifractal spectra of the tachograms of healthy subjects have curvature values around the maxima (*q* = 0) that are much lower than the curvature values around the maximum of seen for CHF patients. On the other hand, most of the multifractal spectra of the tachograms of healthy subjects tend to be right skewed; in the 24 h tachograms, 49 of 54 are right skewed, and in those of 6 h awake, 45 of 54 are right skewed and in those of 6 h asleep, all the spectra are right skewed. The parameters *r* and *K* can complement each other, as mentioned in the article (see Figure 8), there are several spectra of healthy people whose spectra are a little charged to the left, but their curvature around the maximum is small, in the same order as the curvature average of all healthy subjects.

The curvature *K* around the maxima of the spectra is another parameter that we introduced because we have noted that the multifractal spectra of the CHF patients are spiky around the maxima. The average values of the curvature maxima of the 24 h and 6 h interbeat time series (when the healthy persons and CHF patients are asleep or awake) show a difference between their curvature values; that is, the average curvature values are bigger in CHF patients than in healthy persons and these values increase with the severity of the damage in the heart as a result of the CHF condition. Amaral et al. [4] reported that the width of the multifractal spectra decreases when a beta-blocking drug is administered to healthy subjects producing a diminution of the effects of the sympathetic branch of the neuroautonomic control mechanisms and they found that the width of the spectra decreased, although the healthy condition was recovered after a certain time interval. That is, seemingly the CHF spectra reflect a dynamical behaviour analogous to the case with the blocking of the sympathetic branch. These authors also reported an experiment with a drug that blocks the parasympathetic branch with more dramatic results in the dynamic behaviour of the heart (see Figure 3b of reference [4]); that is similar to the observed spectra of the more severe cases of CHF patients. Our results have no inconsistencies with the very important findings of Amaral et al. [4], because we have found that the curvature around the maxima is growing with the severity of the CHF condition in the NYHA III-IV cases where there is a severe impairment of the parasympathetic branch. On the other hand, with the stochastic models of references [2,30], we obtained (see Figure 6 of reference [20]) a reasonable reproduction of multifractal spectra of both healthy and CHF cases including the narrowing of CHF spectra, and some clues of the asymmetry properties of multifractal spectra of healthy subjects and CHF patients reported in the present article. In addition, curvature helps us to discriminate each NYHA classification. In conclusion, the *r* symmetry parameter and the curvature *K* parameter calculated around the multifractal spectra maxima can give us information about the heart health conditions in many people.

## Figures and Tables

**Table 1:** The average index of symmetry *r*.

	Healthy	CHF
24 h RR time series.	Right skewed (49 subjects) *r* = 1.9 ± 1.0	Right skewed (25 patients) *r* = 1.5 ± 0.5
	Left skewed (5 subjects) *r* = 0.9 ± 0.05	Left skewed (19 patients) *r* = 0.8 ± 0.16
6 h RR time series asleep	Right skewed (54 subjects) *r* = 4.5 ± 5.3	Right skewed (25 patients) *r* = 1.9 ± 0.8
	Left skewed (0 subjects)	Left skewed (19 patients) *r* = 0.7 ± 0.18
6 h RR time series awake	Right skewed (45 subjects) *r* = 2.0 ± 0.8	Right skewed (31 patients) *r* = 1.9 ± 0.8
	Left skewed (9 subjects) *r* = 0.8 ± 0.08	Left skewed (9 patients) *r* = 0.7 ± 0.2

**Table 2:** The *r* values of healthy people time series of 24 h. The acronym in parentheses refers to age in years and gender (female or male), nsr means normal sinus rhythm (for healthy persons).

Person	*r*	Person	*r*	Person	*r*
nsr01(64F)	1.85	nsr19(65F)	1.69	nsr37(63M)	2.56
nsr02(67M)	1.52	nsr20(58F)	1.14	nsr38(62M)	1.23
nsr03(67F)	1.14	nsr21(59M)	2.19	nsr39(70F)	1.49
nsr04(62F)	2.05	nsr22(68M)	3.38	nsr40(63F)	3.34
nsr05(62F)	1.80	nsr23(66F)	1.97	nsr41(64F)	1.28
nsr06(64M)	2.66	nsr24(63F)	2.07	nsr42(68F)	2.21
nsr07(76M)	2.12	nsr25(75M)	0.92	nsr43(66M)	1.87
nsr08(64F)	1.76	nsr26(72M)	0.83	nsr44(65F)	1.57
nsr09(66M)	1.22	nsr27(64M)	4.47	nsr45(67F)	1.76
nsr10(61F)	1.71	nsr28(65M)	2.20	nsr46(63F)	1.82
nsr11(65F)	1.04	nsr29(63M)	1.44	nsr47(28.5M)	1.01
nsr12(66M)	2.94	nsr30(70F)	6.65	nsr48(38M)	0.88
nsr13(63F)	1.34	nsr31(67M)	2.35	nsr49(39M)	1.06
nsr14(65F)	1.40	nsr32(68M)	1.81	nsr50(29M)	0.83
nsr15(74M)	1.34	nsr33(65M)	3.93	nsr51(40M)	1.15
nsr16(73F)	1.24	nsr34(67M)	2.75	nsr52(39M)	1.05
nsr17(71F)	1.40	nsr35(66M)	1.18	nsr53(35M)	1.69
nsr18(68M)	1.27	nsr36(60F)	1.24	nsr54(35M)	0.94

**Table 3:** The *r* values for 6 h time series of healthy people and CHF patients awake and asleep, CHF means congestive heart failure (for diseased persons).

Person	Awake	Asleep	Patient	Awake	Asleep
nsr01	1.88	3.17	chf001	1.45	1.56
nsr02	1.52	2.90	chf002	1.06	0.62
nsr03	1.19	3.01	chf004	1.44	1.16
nsr04	1.58	4.09	chf005	0.63	1.43
nsr05	0.72	2.99	chf006	No data	1.85
nsr06	0.92	3.09	chf007	2.91	1.06
nsr07	1.34	20.70	chf008	3.67	1.94
nsr08	2.28	3.36	chf010	1.04	0.28
nsr09	1.41	3.99	chf011	1.70	1.51
nsr10	1.04	3.06	chf012	1.02	1.20
nsr11	0.95	3.10	chf013	0.30	1.02
nsr12	3.05	3.19	chf014	0.89	1.01
nsr13	1.61	2.46	chf015	0.62	1.05
nsr14	1.47	2.86	chf201	2.70	1.15
nsr15	0.95	31.86	chf202	0.80	0.70
nsr16	0.83	1.65	chf203	2.84	3.71
nsr17	1.03	1.57	chf204	2.13	1.60
nsr18	2.15	1.45	chf205	0.61	0.47
nsr19	2.06	2.32	chf207	0.75	1.28
nsr20	1.32	1.45	chf208	2.56	1.62
nsr21	1.01	2.38	chf209	No data	0.80
nsr22	3.10	3.67	chf210	2.45	2.01
nsr23	1.14	1.74	chf211	0.82	2.51
nsr24	4.15	1.55	chf212	0.44	0.66
nsr25	0.79	1.85	chf213	0.89	2.65
nsr26	0.75	8.41	chf214	0.98	1.51
nsr27	2.41	2.80	chf215	2.45	0.75
nsr28	2.30	3.50	chf216	2.48	1.89
nsr29	1.05	2.92	chf217	1.30	3.63
nsr30	2.53	17.01	chf218	0.82	3.28
nsr31	1.82	2.73	chf219	1.10	2.20
nsr32	1.18	3.40	chf220	0.91	1.25
nsr33	3.59	6.52	chf221	0.82	2.18
nsr34	1.93	9.43	chf223	0.64	1.09
nsr35	1.94	1.49	chf224	1.56	2.60
nsr36	3.52	4.53	chf225	0.68	0.98
nsr37	3.80	3.16	chf226	2.45	3.17
nsr38	1.98	2.40	chf227	1.20	2.56
nsr39	2.81	3.48	chf228	1.12	3.01
nsr40	2.57	5.39	chf229	0.53	0.69
nsr41	1.69	1.22			
nsr42	2.12	1.40			
nsr43	3.22	3.07			
nsr44	2.14	2.45			
nsr45	2.17	4.09			
nsr46	1.05	3.73			
nsr47	1.85	2.87			
nsr48	0.86	2.91			
nsr49	1.14	14.96			
nsr050	1.25	2.76			
nsr051	1.52	3.88			
nsr052	1.28	3.09			
nsr053	2.52	2.91			
nsr054	0.86	3.86			

**Table 4:** The *r* values of CHF patients time series of 24 h. In parenthesis, we have NYHA index, age in years and gender (M is for male, F is for female, U is for unknown).

Patient	*r*	Patient	*r*	Patient	*r*
chf001(III-IV, 71M)	1.09	chf201(III, 55M)	1.51	chf216(II, 58U)	0.94
chf002(III-IV, 61F)	1.05	chf202(III, 59F)	0.85	chf217(I, 50U)	1.99
chf003(III-IV, 63M)	1.73	chf203(III, 68M)	1.25	chf218(I, 72U)	1.04
chf004(III-IV, 54M)	1.18	chf204(III, 62M)	1.62	chf219(III, 62U)	1.39
chf005(III-IV, 59F)	0.72	chf205(III, 39M)	0.47	chf220(II, 64U)	1.02
chf006(III-IV, UM)	1.89	chf206(III, 38F)	0.91	chf221(I, 37U)	1.11
chf007(III-IV, 48M)	2.41	chf207(III, 62M)	0.82	chf222(III, 63U)	0.76
chf008(III-IV, 51M)	2.51	chf208(III, 62M)	0.77	chf223(III, 56U)	0.87
chf009(III-IV, 63F)	0.87	chf209(III, 65M)	0.88	chf224(II, 35U)	1.85
chf010(III-IV, 22M)	1.03	chf210(III, 43M)	2.02	chf225(III, 66U)	0.78
chf011(III-IV, 54F)	1.54	chf211(II, 34U)	1.48	chf226(II, 51U)	2.44
chf012(III-IV, 61M)	0.89	chf212(II, 54U)	0.41	chf227(III, 64U)	1.27
chf013(III-IV, 63M)	0.71	chf213(I, 53U)	1.34	chf228(III, 31U)	1.25
chf014(III-IV, 61M)	1.01	chf214(II, 79U)	0.89	chf229(III,58U)	0.54
chf015(III-IV, 53M)	0.60	chf215(II, 43U)	0.86		

**Table 5:** Average curvature maxima values of 6 h in CHF patients when they are awake and asleep for each NYHA classification.

Time Series of 6 h	Awake	Asleep
NYHA I	325 ± 57	706 ± 131
NYHA II	1434 ± 270	1442 ± 276
NYHA III	1649 ± 304	1651 ± 300
NYHA III-IV	3419 ± 890	3054 ± 801
healthy people	587 ± 108	1177 ± 220
CHF patients	1986 ± 380	1971 ± 377
